# Supplementary material for: Comparative Study of the Steroidogenic Effects of Human Chorionic Gonadotropin and Thieno[2,3-D]pyrimidine-Based Allosteric Agonist of Luteinizing Hormone Receptor in Young Adult, Aging and Diabetic Male Rats
Source: Int J Mol Sci. 2020 Oct 11;21(20):7493. doi: 10.3390/ijms21207493 (PMC7590010; doi:10.3390/ijms21207493)
Supplement: Supplementary file 1 [file ijms-21-07493-s001.zip › Supplementary files/Fig. S1 r1.pdf]

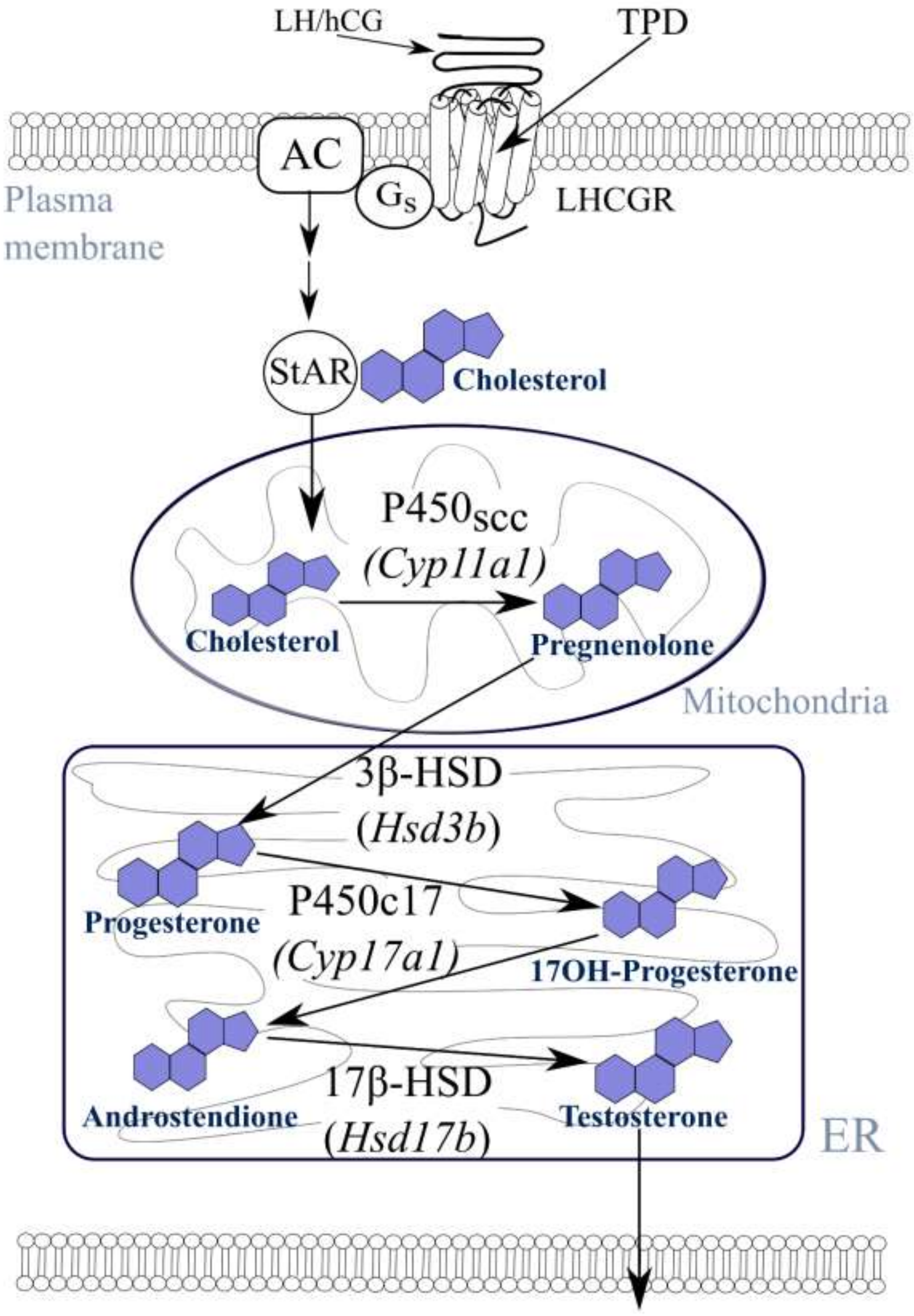

Abbreviations: LH – luteinizing hormone; hCG – human chorionic gonadotropin; TPD – thieno[2,3-*d*]pyrimidine derivative; LHCGR – LH/hCG receptor; G_s – heterotrimeric G_s protein; AC – adenylyl cyclase; StAR – steroidogenic acute regulatory protein; $P450_{scc}$ – cytochrome 450, cholesterol side-chain cleavage enzyme (CYP11A1); 3β-HSD – 3β-hydroxysteroid dehydrogenase/Δ5-4 isomerase; P450c17 – cytochrome P450 17A1/steroid 17α-monooxygenase (CYP17A1); 17β-HSD – 17β-hydroxysteroid dehydrogenase; 17OH-progesterone – 17-hydroxyprogesterone.
