# Supplementary material for: Comparative Study of the Steroidogenic Effects of Human Chorionic Gonadotropin and Thieno[2,3-D]pyrimidine-Based Allosteric Agonist of Luteinizing Hormone Receptor in Young Adult, Aging and Diabetic Male Rats
Source: Int J Mol Sci. 2020 Oct 11;21(20):7493. doi: 10.3390/ijms21207493 (PMC7590010; doi:10.3390/ijms21207493)
Supplement: Supplementary file 1 [file ijms-21-07493-s001.zip › Supplementary files/Fig. S2 r1.pdf]

**A** 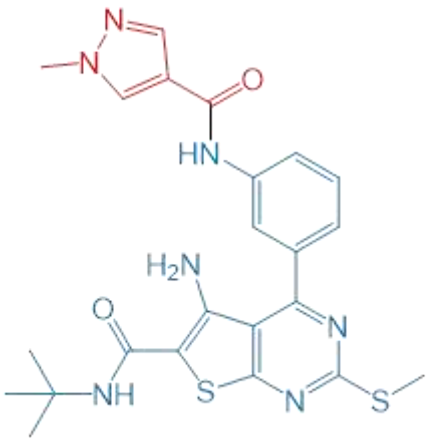	
**B** 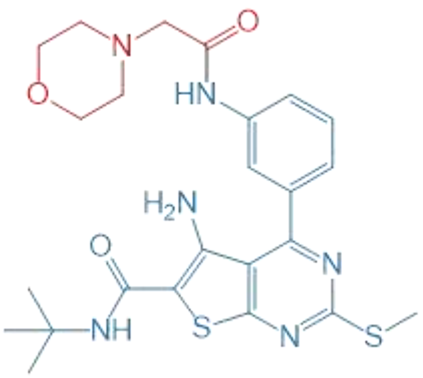	

A - 5-amino-*N*-*tert*-butyl-4-(3-(1-methylpyrazole-4-carboxamido)phenyl)-2-(methylthio)thieno[2,3-*d*]pyrimidine-6-carboxylic acid amide (TP4)
B - Org 43553
