# Supplementary material for: Comparative Study of the Steroidogenic Effects of Human Chorionic Gonadotropin and Thieno[2,3-D]pyrimidine-Based Allosteric Agonist of Luteinizing Hormone Receptor in Young Adult, Aging and Diabetic Male Rats
Source: Int J Mol Sci. 2020 Oct 11;21(20):7493. doi: 10.3390/ijms21207493 (PMC7590010; doi:10.3390/ijms21207493)
Supplement: Supplementary file 1 [file ijms-21-07493-s001.zip › Supplementary files/Fig. S5 r1.pdf]

A

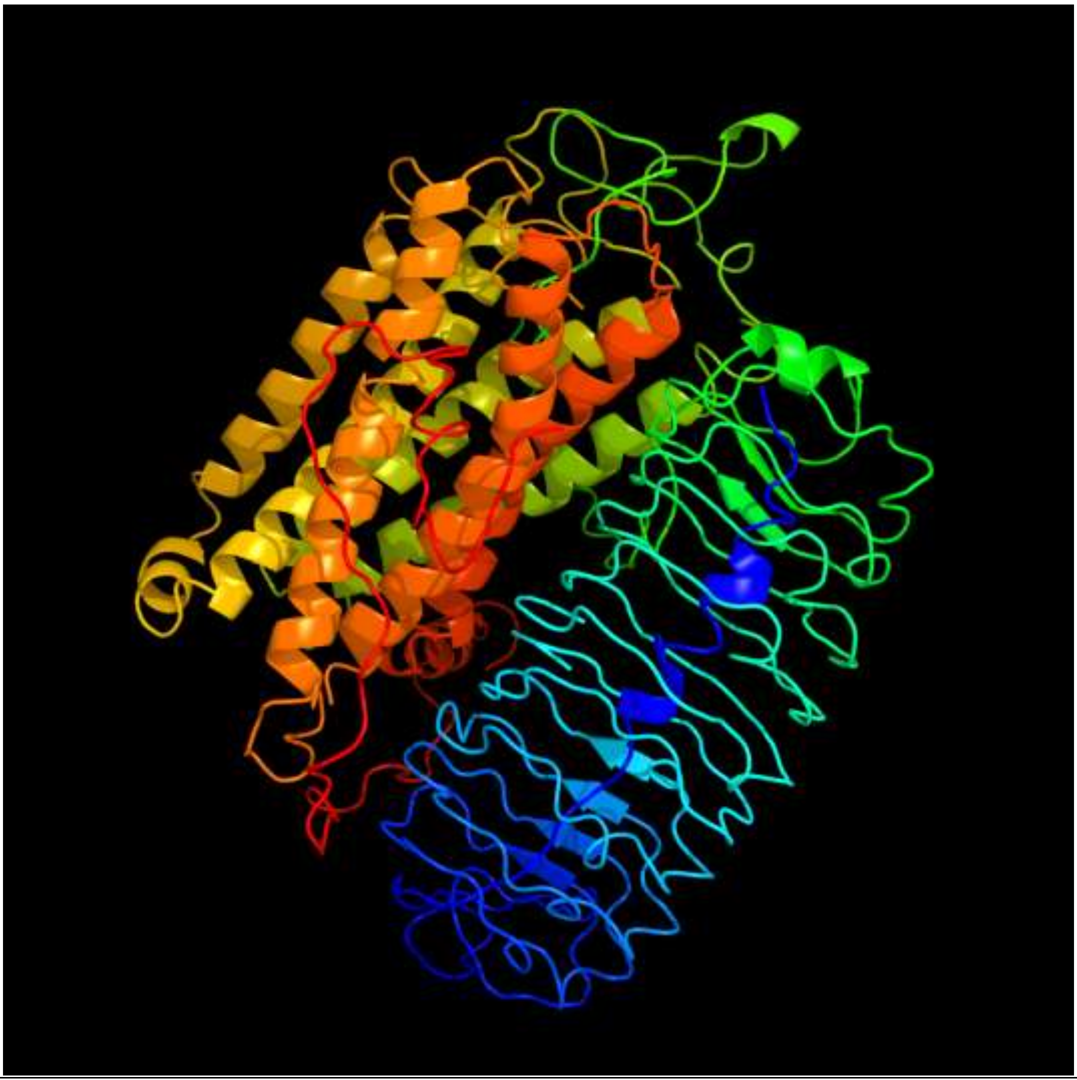

B

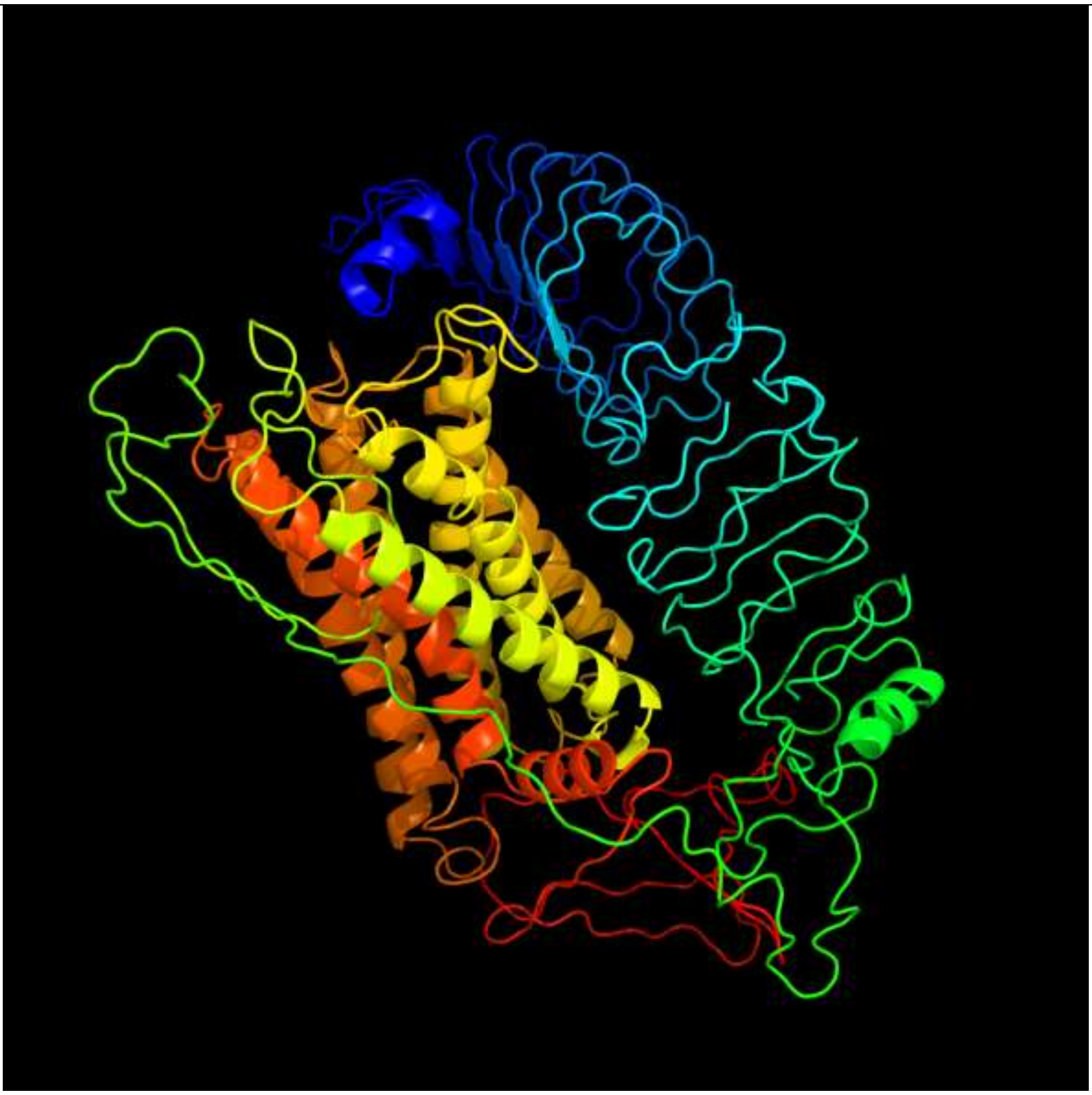

Three-dimensional structures of full-length rat luteinizing hormone/chorionic gonadotropin (LHCGR) and thyroid-stimulating hormone (TSHR) receptors generated by homology modeling. Protein secondary structure elements and folding is based on the method of parallel annealing with replica exchange. The transmembrane fragment is built on the basis of the transmembrane domain of bovine rhodopsin. For modeling, 13 and 10 template models were used, respectively.
