# Supplementary material for: Comparative Study of the Steroidogenic Effects of Human Chorionic Gonadotropin and Thieno[2,3-D]pyrimidine-Based Allosteric Agonist of Luteinizing Hormone Receptor in Young Adult, Aging and Diabetic Male Rats
Source: Int J Mol Sci. 2020 Oct 11;21(20):7493. doi: 10.3390/ijms21207493 (PMC7590010; doi:10.3390/ijms21207493)
Supplement: Supplementary file 1 [file ijms-21-07493-s001.zip › Supplementary files/Table S1 r1.pdf]

| Group of rats | Basal | Forskolin, $10^{-5}$ M | GppNHp, $10^{-5}$ M |
|---|---|---|---|
| | Adenylyl cyclase activity, pmol cAMP/min/mg of membrane protein, *M* ± *SEM* | | |
| Young | 26.1 ± 1.1 | 267.5 ± 3.9 | 159.2 ± 3.5 |
| Aging | 22.8 ± 1.2[b] | 254.2 ± 7.0[b] | 133.4 ± 6.9[a, b] |
| Diab | 18.5 ± 0.9[a] | 197.9 ± 5.3[a] | 92.2 ± 6.1[a] |

*Notes.* [a] - the difference between the young rats and the aging or diabetic rats is significant at $p < 0.05$; [b] - the difference between the aging and diabetic rats is significant at $p < 0.05$. The data are presented as the *M* ± *SEM*, *n*=5.
