# Supplementary material for: Comparative Study of the Steroidogenic Effects of Human Chorionic Gonadotropin and Thieno[2,3-D]pyrimidine-Based Allosteric Agonist of Luteinizing Hormone Receptor in Young Adult, Aging and Diabetic Male Rats
Source: Int J Mol Sci. 2020 Oct 11;21(20):7493. doi: 10.3390/ijms21207493 (PMC7590010; doi:10.3390/ijms21207493)
Supplement: Supplementary file 1 [file ijms-21-07493-s001.zip › Supplementary files/Table S2 r1.pdf]

	Basal	Forskolin, 10^{-5} M	GppNHp, 10^{-5} M	TSH, 10^{-9} M
	Adenylyl cyclase (AC) activity, pmol cAMP/min/mg of membrane protein, $M \pm SEM$			
- TP4/2	19.0 ± 1.3	413.5 ± 9.1 [a]	184.1 ± 5.6 [a]	138.7 ± 1.8 [a]
+ TP4/2, 10^{-5} M	18.3 ± 0.8	427.7 ± 13.9 [a]	176.2 ± 7.3 [a]	135.3 ± 5.8 [a]

Notes. [a] - the difference between the basal adenylyl cyclase activity and the forskolin-, GppNHp- and TSH-stimulated adenylyl cyclase activity is significant at $p < 0.05$. The data are presented as the $M \pm SEM$, n=5.
