# Supplementary material for: Comparative Study of the Steroidogenic Effects of Human Chorionic Gonadotropin and Thieno[2,3-D]pyrimidine-Based Allosteric Agonist of Luteinizing Hormone Receptor in Young Adult, Aging and Diabetic Male Rats
Source: Int J Mol Sci. 2020 Oct 11;21(20):7493. doi: 10.3390/ijms21207493 (PMC7590010; doi:10.3390/ijms21207493)
Supplement: Supplementary file 1 [file ijms-21-07493-s001.zip › Supplementary files/Table S3 r1.pdf]

	10-00	12-30	14-00	$AUC_{10.00\text{-}14.00}$
**A (**fT4**)**				
Control	18.7 ± 0.7	19.9 ± 1.0	18.1 ± 0.8	76.6 ± 3.1
Control+TP4/2	19.5 ± 0.9	20.2 ± 1.5	18.6 ± 1.3	78.7 ± 3.8
Control+TRH	20.4 ± 1.3	27.9 ± 1.3 [a]	32.9 ± 1.7 [a]	106.0 ± 3.5 [b]
Control+TP4/2+TRH	19.1 ± 0.8	27.6 ± 1.3 [a]	31.7 ± 1.6 [a]	102.9 ± 3.6 [b]
**B (**tT4**)**				
Control	95.1 ± 2.5	96.2 ± 2.1	92.3 ± 2.1	380.6 ± 7.8
Control+TP4/2	94.5 ± 3.0	97.2 ± 3.5	94.3 ± 3.1	383.1 ± 9.5
Control+TRH	93.0 ± 4.3	108.3 ± 3.4 [a]	121.2 ± 3.9 [a]	423.8 ± 10.0 [b]
Control+TP4/2+TRH	93.2 ± 2.4	106.8 ± 2.5 [a]	122.3 ± 4.8 [a]	421.7 ± 7.6 [b]
**C (**tT3**)**				
Control	2.6 ± 0.1	2.5 ± 0.1	2.3 ± 0.2	10.00± 0.38
Control+TP4/2	2.6 ± 0.1	2.5 ± 0.2	2.4 ± 0.2	9.97 ± 0.41
Control+TRH	2.5 ± 0.1	2.9 ± 0.1	3.9 ± 0.3	11.80 ± 0.17 [b]
Control+TP4/2+TRH	2.5 ± 0.2	2.8 ± 0.2 [a]	3.8 ± 0.3 [a]	11.64 ± 0.57

Note. TP4/2 was administered into male rats (i.p., 20 mg/kg, in 200 μL of DMSO), and the control group was injected with DMSO (i.p., 200 μL), a solvent for TP4/2. Thyrotropin-releasing hormone (TRH) ("Sigma-Aldrich", USA) was administered intranasally (300 μg/kg, in 20 μL of saline). The first blood sampling was obtained at 10.00. At 10.30 the groups Control+TP4/2 and Control+TP4/2+TRH were injected with TP4/2, and the groups Control and Control+TRH were treated with DMSO. At 11.00 the groups Control+TRH and Control+TP4/2+TRH were treated with intranasally administered TRH, and the groups Control and Control+TP4/2 were treated with intranasally administered saline instead of TRH. At 12.30 and 14.00 the second and third blood samples were obtained (*n*=5 in each group). The blood samples were obtained from the tail vein using a local anesthesia (2% lidocaine solution, 2–4 mg/kg). The blood levels of free (fT4) and total (tT4) thyroxine and total triiodothyronine (tT3) were determined using the ELISA kits obtained from "Immunotech" (Russia). [a] - the difference between the levels of thyroid hormones at 10.00 and their levels at 12.30 and 14.00 is significant at $p < 0.05$; [b] - the difference between the control and TRH-treated rats is significant at $p < 0.05$. The data are presented as the $M \pm SEM$, n=5.
