# Supplementary material for: Comparative Study of the Steroidogenic Effects of Human Chorionic Gonadotropin and Thieno[2,3-D]pyrimidine-Based Allosteric Agonist of Luteinizing Hormone Receptor in Young Adult, Aging and Diabetic Male Rats
Source: Int J Mol Sci. 2020 Oct 11;21(20):7493. doi: 10.3390/ijms21207493 (PMC7590010; doi:10.3390/ijms21207493)
Supplement: Supplementary file 1 [file ijms-21-07493-s001.zip › Supplementary files/Table S4 r1.pdf]

Group	10-00	11-00	13-00	15-00	$AUC_{10.00-15.00}$ (total)
**TP4/2**					
**A)** Intraperitoneal administration of TP4/2 at the doses of 5-50 mg/kg					
C+DMSO (i.p.)	14.2 ± 2.2	18.7 ± 4.1	20.5 ± 2.1	19.5 ± 3.1	95 ± 14
C+TP, 5 mg/kg	15.4 ± 3.0	25.5 ± 2.2	27.7 ± 2.7	28.0 ± 1.5	129 ± 8
C+TP, 10 mg/kg	17.3 ± 2.3	27.7 ± 1.4	37.6 ± 3.2	38.8 ± 5.7	164 ± 13
C+TP, 15 mg/kg	18.6 ± 2.4	34.5 ± 3.6	58.4 ± 5.6	60.2 ± 9.4	238 ± 23
C+TP, 25 mg/kg	17.8 ± 4.1	41.0 ± 4.7	76.7 ± 8.9	77.6 ± 9.8	301 ± 18
C+TP, 50 mg/kg	14.3 ± 2.6	42.6 ± 7.1	79.7 ± 4.0	76.3 ± 1.4	307 ± 15
**B)** Subcutaneous administration of TP4/2 at the doses of 5-50 mg/kg					
C+DMSO (s.c.)	16.6 ± 2.9	16.5 ± 4.0	17.2 ± 2.4	14.9 ± 3.8	82 ± 16
C+TP, 5 mg/kg	16.0 ± 3.9	15.2 ± 4.4	18.7 ± 3.9	17.2 ± 5.6	83 ± 19
C+TP, 10 mg/kg	13.9 ± 3.2	14.9 ± 2.7	18.8 ± 5.1	22.5 ± 6.7	90 ± 21
C+TP, 15 mg/kg	12.3 ± 3.4	14.9 ± 2.9	27.4 ± 6.0	29.5 ± 7.0	110 ± 22
C+TP, 25 mg/kg	15.1 ± 3.6	18.5 ± 3.9	30.2 ± 8.1	30.5 ± 9.2	128 ± 26
C+TP, 50 mg/kg	15.9 ± 3.9	20.6 ± 3.9	29.8 ± 5.1	29.2 ± 3.0	131 ± 15
**Human chorionic gonadotropin**					
**C)** Intraperitoneal administration of hCG at the doses of 5-100 IU/rat					
C+saline (i.p.)	17.7 ± 4.6	15.4 ± 3.1	18.1 ± 2.9	17.0 ± 2.7	85 ± 15
C-CG, 5 IU/rat	12.8 ± 2.9	16.8 ± 2.7	49.0 ± 9.2	57.8 ± 9.6	189 ± 28
C-CG, 10 IU/rat	12.9 ± 3.3	24.2 ± 4.9	75.8 ± 9.4	59.2 ± 8.1	254 ± 33
C-CG, 20 IU/rat	12.6 ± 3.0	37.1 ± 7.0	78.0 ± 4.3	58.5 ± 9.6	279 ± 4
C-CG, 50 IU/rat	13.3 ± 2.3	65.1 ± 7.4	102.8 ± 19.9	70.9 ± 6.9	381 ± 36
C-CG, 100 IU/rat	13.6 ± 2.8	70.9 ± 16.5	79.3 ± 11.0	59.8 ± 9.8	333 ± 47
**D)** Subcutaneous administration of hCG at the doses of 5-100 IU/rat					
C+saline (s.c.)	14.2 ± 2.2	18.7 ± 4.1	20.5 ± 2.1	19.5 ± 3.1	95 ± 14
C-CG, 5 IU/rat	12.0 ± 2.4	25.7 ± 2.7	43.0 ± 6.6	42.4 ± 5.7	173 ± 22
C-CG, 10 IU/rat	12.8 ± 2.6	43.8 ± 8.6	89.4 ± 11.7	73.7 ± 9.2	324 ± 46
C-CG, 20 IU/rat	18.1 ± 5.2	71.5 ± 15.7	113.0 ± 12.5	94.1 ± 16.7	436 ± 63
C-CG, 50 IU/rat	16.2 ± 1.6	76.2 ± 9.4	158.6 ± 14.6	118.5 ± 15.2	558 ± 33
C-CG, 100 IU/rat	12.6 ± 2.1	81.4 ± 4.1	167.1 ± 10.9	111.4 ± 18.9	574 ± 35

Note. The drugs or saline or DMSO were administered at 10.00. *n*=3.
