# Supplementary material for: Comparative Study of the Steroidogenic Effects of Human Chorionic Gonadotropin and Thieno[2,3-D]pyrimidine-Based Allosteric Agonist of Luteinizing Hormone Receptor in Young Adult, Aging and Diabetic Male Rats
Source: Int J Mol Sci. 2020 Oct 11;21(20):7493. doi: 10.3390/ijms21207493 (PMC7590010; doi:10.3390/ijms21207493)
Supplement: Supplementary file 1 [file ijms-21-07493-s001.zip › Supplementary files/Table S5 r1.pdf]

Genes	Forward/Reverse sequence	Product size (bp)	Annealing temperature (°C)	Genbank
Lhr	(For) CTGCGCTGTCCTGGCC	103	55	NM_012978.1
	(Rev) CGACCTCATTAAGTCCCCTGAA			
Star	(For) AAGGCTGGAAGAAGGAAAGC	66	55	NM_031558.3
	(Rev) CACCTGGCACCACCTTACTT			
Cyp11a1	(For) TATTCCGCTTTGCCTTTGAG	74	55	NM_017286.3
	(Rev) CACGATCTCCTCCAACATCC			
Hsd3B	(For) AGGCCTGTGTCCAAGCTAGTGT	161	55	XM_017591325.1
	(Rev) CTCGGCCATCTTTTTGCTGTAT			
Cyp17a1	(For) CATCCCCCACAAGGCTAAC	61	55	XM_006231435.3
	(Rev) TGTGTCCTTGGGGACAGTAAA			
Hsd17B	(For) CCTTTGGCTTTGCCATGAGA	68	55	NM_024392.2
	(Rev) CAATCCATCCTGCTCCAACCT			
Actb	(For) CTGGCACCACACCTTCTACA	125	55	NM_031144.3
	(Rev) AGGTCTCAAACATGATCTGGGT			
