# Supplementary figures and images for: Comparative Study of the Steroidogenic Effects of Human Chorionic Gonadotropin and Thieno[2,3-D]pyrimidine-Based Allosteric Agonist of Luteinizing Hormone Receptor in Young Adult, Aging and Diabetic Male Rats
Source: Int J Mol Sci. 2020 Oct 11;21(20):7493. doi: 10.3390/ijms21207493 (PMC7590010; doi:10.3390/ijms21207493)

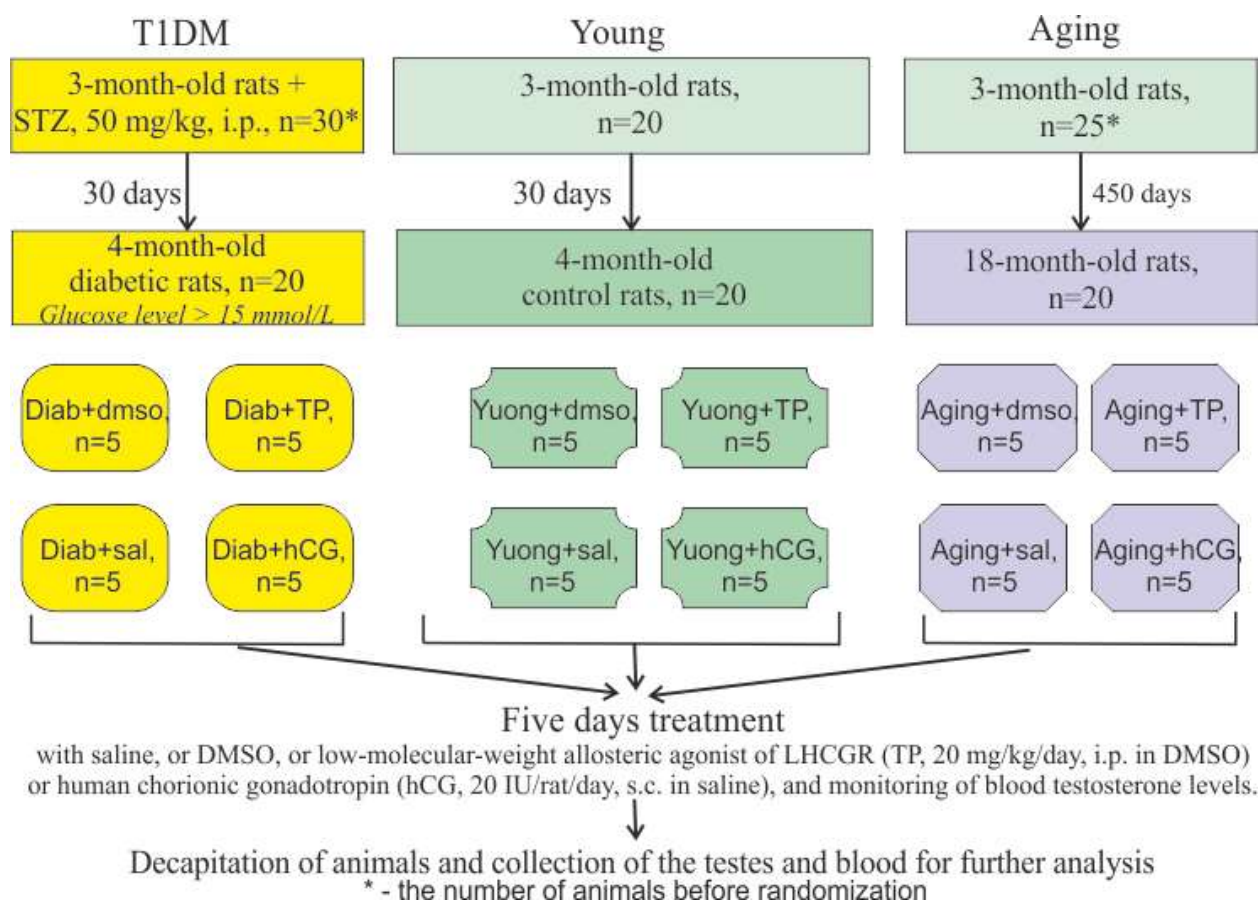

Supplement: Supplementary file 1 [file ijms-21-07493-s001.zip › Supplementary files/Fig. S3 r1.pdf]

**A**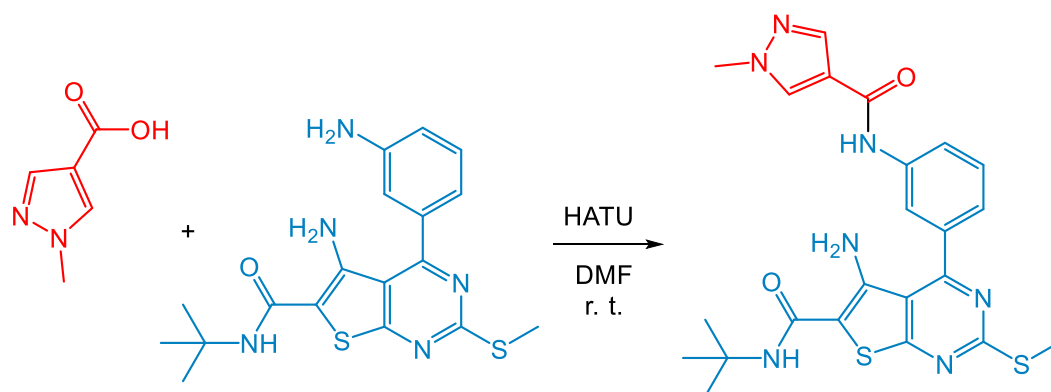**B**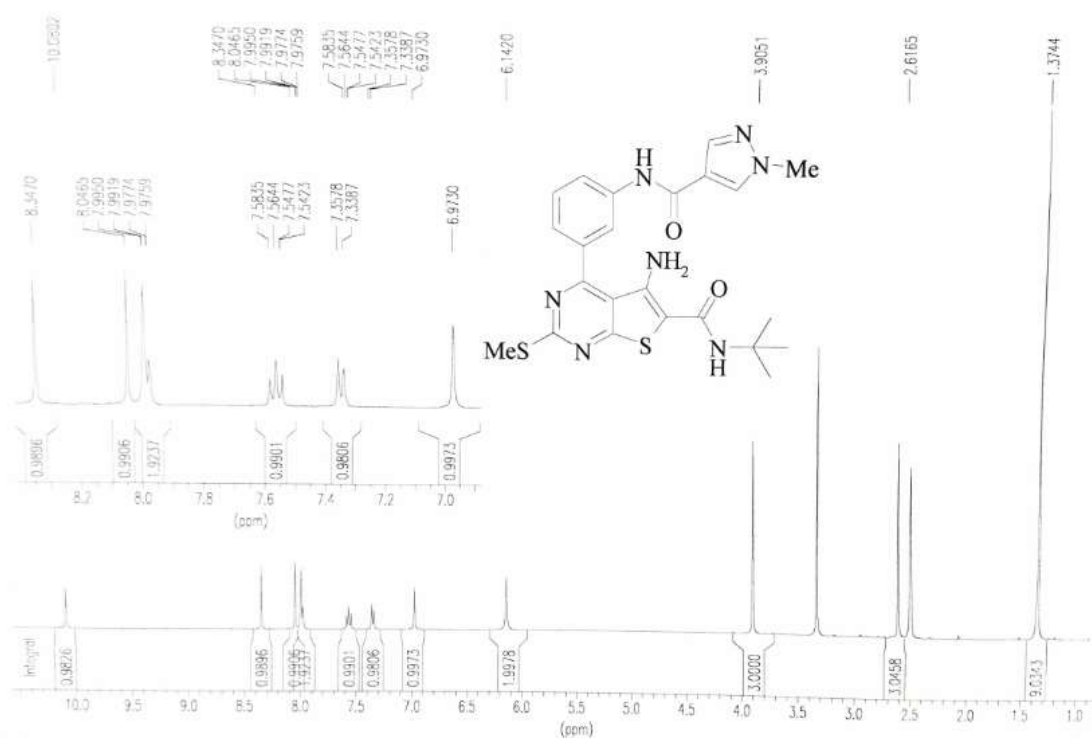**C**

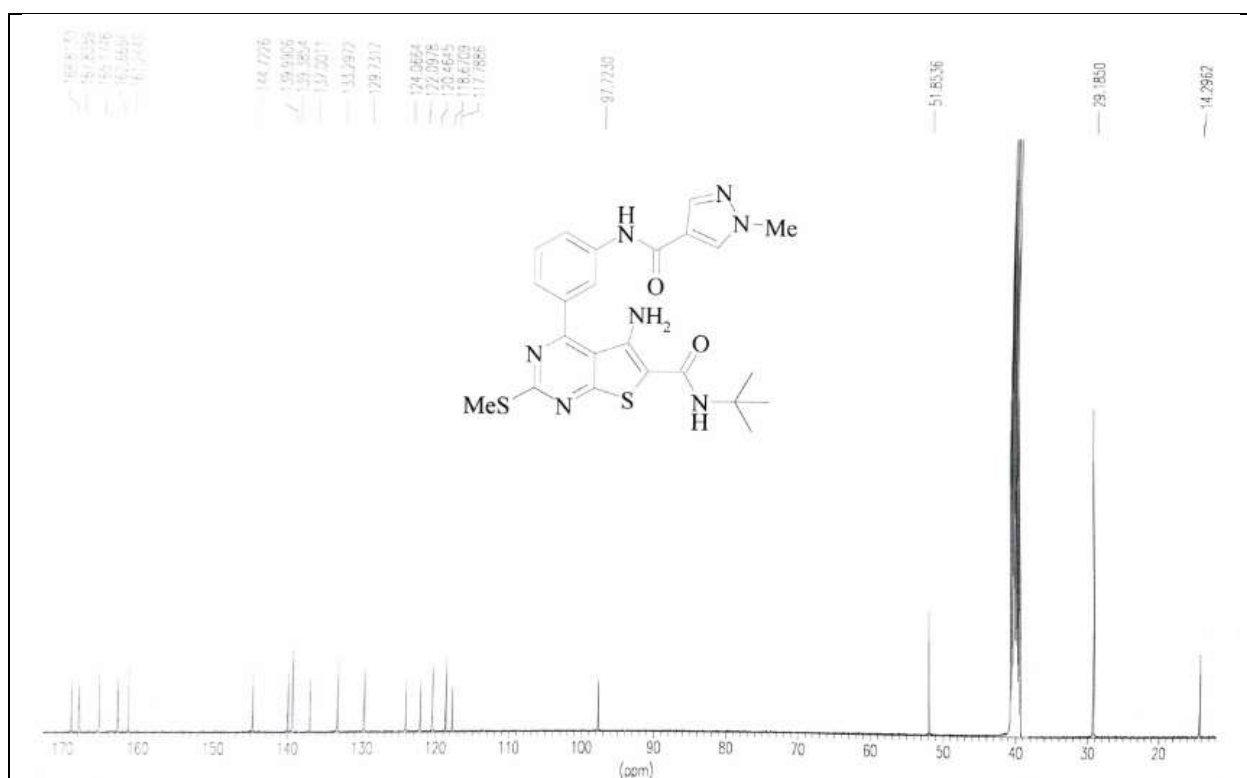

Supplement: Supplementary file 1 [file ijms-21-07493-s001.zip › Supplementary files/Fig. S4 r1.pdf]
